# Age-specific familial risks in cancer as clues to germline genetic and environmental causes: focus on colorectal, endometrial, prostate, kidney, breast and lung cancers

**DOI:** 10.1186/s13053-024-00301-8

**Published:** 2025-02-21

**Authors:** Kari Hemminki, Asta Försti, Otto Hemminki, Rodney J. Scott, Akseli Hemminki

**Affiliations:** 1 Biomedical Center, Faculty of Medicine, Charles University, Pilsen, 30605 Czech Republic; 2 Division of Cancer Epidemiology, German Cancer Research Center (DKFZ), Im Neuenheimer Feld 580, Heidelberg, 69120 Germany; 3 Hopp Children’s Cancer Center (KiTZ), Heidelberg, Germany; 4 Division of Pediatric Neurooncology, German Cancer Research Center (DKFZ), Heidelberg, Germany; 5 Cancer Consortium (DKTK), Heidelberg, Germany; 6 Department of Urology, Helsinki University Hospital, Helsinki, Finland; 7 Cancer Gene Therapy Group, Translational Immunology Research Program, University of Helsinki, Helsinki, Finland; 8 The University of Newcastle, Callaghan, NSW Australia; 9 Hunter Medical Research Institute, New Lambton, NSW Australia; 10 Comprehensive Cancer Center, Helsinki University Hospital, Helsinki, Finland

**Keywords:** Familial risk, Germline genetics, Heredity, Age of onset, Early onset

## Abstract

**Background:**

The Swedish Family-Cancer Database (FCD) is the largest source of data on familial cancer in the world, including practically complete family structures and individual cancer diagnoses from the high-quality cancer registry. We present a novel application of FCD by analyzing age-specific familial risks and interpreting them through likely causes, such as germline pathogenic variants and/or environmental exposures.

**Main body:**

The basic assumption for this approach is that a discrete familial clustering in a narrow age-interval is not random but may provide causal clues. For this analysis we selected reasonably common cancers to meaningfully scrutinize familial risk through adulthood in which cancers are diagnosed, that included colorectal (CRC) and endometrial cancers, prostate and kidney cancers and breast and lung cancers. The interpretation is based on the literature. The highest familial relative risks for CRC and endometrial cancers were found at ages 40–44 years, matching the peak impact of mismatch repair gene mutations. However endometrial cancer showed also a small early onset component which could not be explained. Age-related familial risks for breast, prostate and kidney cancers also matched data from large-scale sequencing; these included the early onset component in kidney cancer which was likely due to *VHL* mutations. Age distribution of familial lung cancer was unique in showing a wide peak extending from middle to old ages, which would be consistent with a combination of direct genetic effects and indirect influence on inheritance of smoking dependence.

**Conclusions:**

The present review of age-specific familial risks and age-of-onset data from the literature may allow an interpretation that the familial and germline landscapes are reasonably harmonious for relatively early onset cancers but at higher ages no discrete peaks can be found which may implicate attenuated impact of high-risk genes and polygenic influence.

## Introduction

Familial cancer can be defined through occurrence of the same cancer in two or more family members. Hereditary cancer has a narrower definition of high-risk familial aggregation, usually through identified constitutional pathogenic variants in predisposing genes. The absence of correlation between spouses for risk of most cancers, particularly of those not related to tobacco smoking or solar exposure, suggests that familial cancers are mainly a result of genetic causes, explained by germline variants [1]. Similar types of correlations between twin or family members are the basis of heritability estimates [2, 3]. The Swedish Family-Cancer Database (FCD) of 16 million individuals is the largest source of data on familial cancer in the world, with primary features of complete family structures and practically complete data on cancers from a high-quality cancer registry [4, 5]. The results are not biased by selection of families or inaccurate reporting of cancer by family members. These premises guarantee the novelty of the obtained results: unbiased familial risk estimates and familial proportions even for rare cancers, by the type and number of familial cases. We have previously considered the relationship between familial risks and the known population impact of the identified cancer susceptibility genes [5]. We discuss below the implications of these findings in terms of germline genetic landscape of familial cancer. Needless to point out that no genotype or environmental data on 16 million people from the late 1800s onward are available.

Here we want to show a novel application of empirical familial risks in the estimation of causes for familial clustering. This is achieved by comparing familial risks by age and assuming that discrete familial clustering in a narrow age-interval has some causal explanation. One prerequisite for this type of analysis is that the numbers of familial cases need to be reasonable to provide statistical support for the assignments. The large sample size of unbiased familial cases is a special advantage of FCD. We thus take examples from FCD to compare age-specific familial (relative) risks (RRs) (called rate ratio in the figures to be shown) between colorectal (CRC) and endometrial cancer; prostate and kidney cancers; and breast and lung cancers, offering the empirical basis for different interpretations. To our knowledge this kind of analysis comparing age-specific familial risks in multiple cancers has not been conducted in other datasets. Some more cancers were included in age-specific analysis in the original publication and in the supplementary material of that publication [4].

When inspecting familial risks in the below figures it is important to note that the curves display the incidence of familial cancer and that of non-familial cancer; familial cases are far fewer and can be estimated from the incidence rates. For this reason, the peak familial risk in the figures is usually at earlier ages (and fewer cases) than the distribution of most familial cases. We show the most common cancer predisposition genes in the figure with an approximate diagnostic age peak (as retrieved from the cited literature), however reminding that the age distributions may be wide and to some extent population dependent. Most of the discussed cancers have a low-risk familial polygenic component that we do not comment on.

## Methods used

Details of the case definitions and methods are found in the original article [4]. Relative risk for cancer was assessed for the 20–84-year-old offspring generation by comparing those with a first-degree family history (through identical cancer) to those without a family history. The incidence rates for the familial and non-familial population were plotted in 5-year age brackets for which rate ratios were calculated with 95% confidence intervals as shown in the subsequent figures.

## CRC and endometrial cancer

**Fig. 1:**
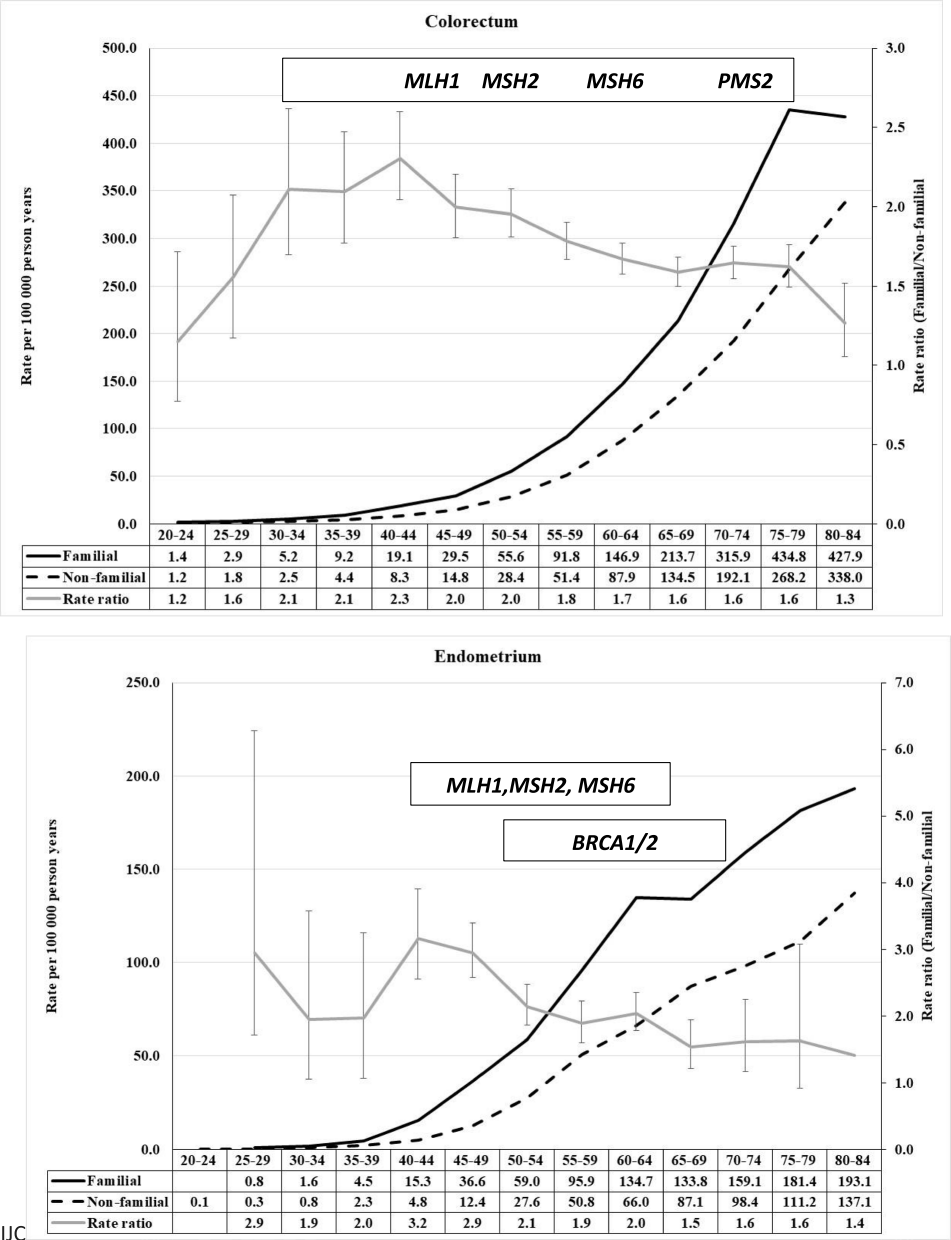
Age-specific incidence rate in population with and without family history of concordant cancer and the rate ratio between the two in colorectal (upper) and endometrial (lower) cancers. Most common cancer predisposition genes are also shown with their approximate diagnostic age peak

Among CRC patients 15.7% were familial in FCD [4]. Figure 1 shows age-specific familial RRs in CRC and endometrial cancers [4]. In CRC the RR increases by age 30–35 years to 2.1 and further by age 40–45 years to 2.3 and then slowly declines to 1.7 by age 75–79 years and finally to 1.3 by age 80–84 years. The most common susceptibility genes in a UK study on newly diagnosed familial early-onset (patient diagnosed before age 56 years) CRC were mismatch repair (MMR) genes accounting for 76% of all identified mutations, *APC* for 11% and *MUTYH* for 7% [6]. *MSH2* and *MLH1* dominated among the four MMR genes (including *MSH6* and *PMS2*) and their mean age of onset was 43 years with a range from early 20 s to 55 years. The mean age of onset for *APC* was 40 and for *MUTYH* 51 years. Results from the prospective Lynch syndrome database agree with the UK data on the most common MMR genes, however the median ages of onset were much higher because the follow-up was to age 75 years, about 50 years for *MLH1*, 56–57 years for *MSH2*, over 60 years for *MSH6* and over 70 years for the rare *PMS2* [7] (Fig. 1). Importantly, the population under study was under regular colonoscopic surveillance [7]. MMR gene mutations were more common in the colon than rectum, and lower number of cases with mutations were found up to age 75 years [7, 8]. While the Swedish family data appear to cover well the diagnostic age-range of the known predisposing genes, the slow decline in familial RR after age 50 years leaves scope for other rare genes and low-risk polygenic inheritance [9].

For endometrial cancer, of which 5.1% was familial, the risk profile shows two peaks, at ages 25–29 years (low case numbers!) and at 40–49 years and a further shoulder at age 60–64 years (Fig. 1) [4]. In addition to *MSH2* and *MSH6, MLH1* also contributes to the risk of endometrial cancer and the median age of onset is over 50 years for all these variants [7]. Thus even the shoulder at over 60 years may be due to MMR genes. The early onset familial clustering that we observed could be due to rare *MLH1* pathogenic variants that have sometimes been diagnosed in young patients [10]. Endometrial cancer is also associated with *BRCA1/2* pathogenic variants [11, 12]. In a Czech study, MMR gene variants were more common than *BRCA1/2* variants with median ages of onset of about 50 and 60 years, respectively [12]. This study also reported *CHEK2* and *ATM* involvement in endometrial cancer families. Endometrial cancer is diagnosed at an estimated frequency of 25% in t Cowden syndrome with frequent germline *PTEN* mutations [11]. The RR may be as high as 13 and median age at onset 31 years [13]. In Sweden, 54 Cowden syndrome-like families were identified with uterine cancer (median diagnostic age 75 years) but no *PTEN* mutations were found [14]. Although the age distribution of familial cases for endometrial cancer is consistent with the age of onset preferences of the known susceptibility genes, the rare early onset component yet awaits an explanation.

## Prostate and kidney cancer

**Fig. 2:**
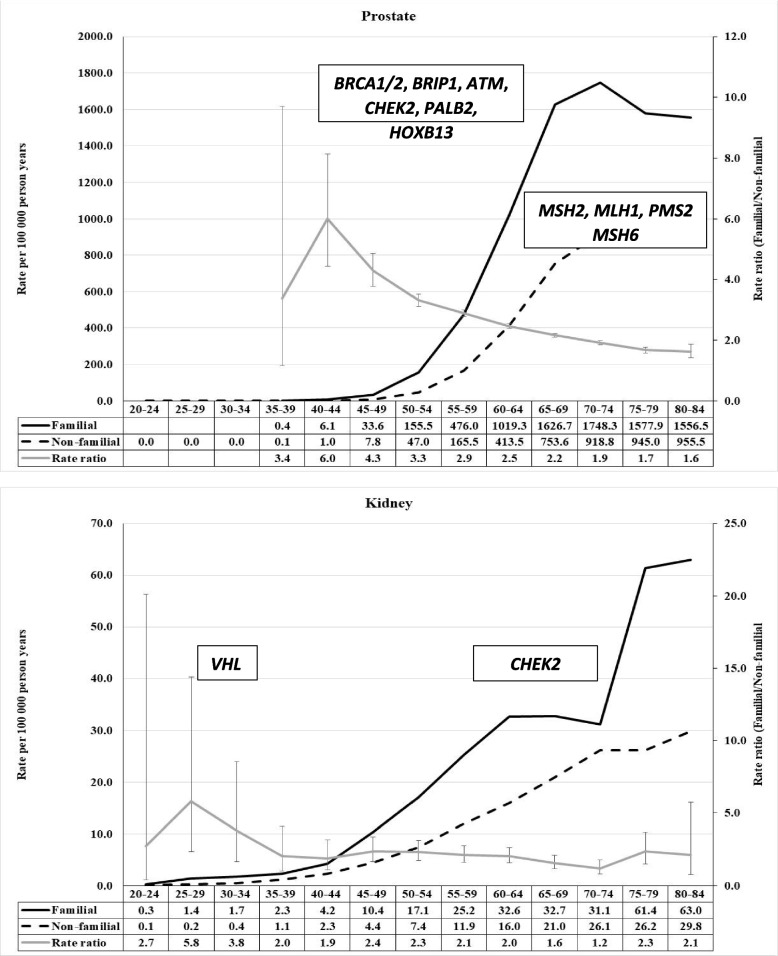
Age-specific incidence rate in population with and without family history of concordant cancer and the rate ratio between the two in prostate (upper) and kidney (lower) cancers. Most common cancer predisposition genes are also shown with their approximate diagnostic age peak

The familial proportion of prostate cancer was 26.4%, by far the highest proportion of all cancers in FCD [4]. Age-specific familial risk profile for prostate cancer was simple, a sharp RR peak of 6.0 at age 40–45 years followed by a smooth decline to RR 1.5 at age 80–84 years (Fig. 2) [4]. It is important to note that the background incidence for prostate cancer is extremely low before age 50 years and larger numbers of familial cases start accumulating by age 60 years. The germline landscape of prostate cancer is dominated by DNA repair genes in the homologous recombination pathway (e.g., *BRCA1, BRCA2, ATM, BRIP1, CHEK2, NBN, BARD1, RAD51C, MRE11A, PALB2* and *FANC* genes) [15]. Other genes include MMR pathway genes and *HOXB13* but the pathogenic variant frequencies were lower than those observed in *BRCA1, ATM* and *CHEK2* [16]. However, MMR mutations are of late onset in prostate cancer and when follow-up was extended to 75 years, a quarter of men were diagnosed with *MSH2* mutations with a median age of about 65 years; only ~ 10% of men carried *MLH1* or *PMS2* pathogenic variants, median age around 70 years [7]. *CHEK2* and *ATM* variants were reported to be enriched in early-onset prostate cancer [17]. According to a US study, pathogenic germline variants identified by hereditary cancer multigene panel testing were revealed in 14.5% of patients; however no data were available for the frequencies of the variants in healthy individuals [16]. The germline pathogenic variant data suggest that deleterious homologous recombination pathway changes dominated in the early onset familial component and the contribution of the MMR genes increased successively towards higher ages when the polygenic influence also contributes (Fig. 2).

Familial proportion of kidney (parenchyma) cancer was 3.8% and the risk profile was characterized by an early onset (age 25–29 years) component, reaching an RR of 5.8, and remaining at the level of over 2.0 up to ~ 80 years of age (Fig. 2) [4]. We have previously identified familial cases of kidney cancer most likely related to VHL mutations [18]. A UK analysis of pathogenic germline variants included 5% of cases with papillary renal cell carcinoma [19]. Among 1336 patients with a mean age of 61 years, 6.4% harbored a pathogenic variant, of which *CHEK2* (40% of all), *ATM, MITF, VHL* and *SDHA* were the most common. No healthy control population was sequenced. The mean age of onset of all pathogenic variants was 59 years compared to 62 years for all patients [19]. The diagnostic age distribution of patients with the pathogenic variants was biphasic with a minor peak (low case numbers!) at 25 years of age and the main broad peak at around 60 years. The mean age of onset for *CHEK2* variant carriers was 65 years and for *VHL* 26 years [19]. *VHL* is one of the genes, like *TP53,* that is related to rare hereditary syndromes but are commonly somatically mutated. In the case of *VHL*, some 50% of sporadic kidney cancers show *VHL* mutations and these have been used to predict the origin of the mutations [20]. Our age-specific profile is in line with the sequencing results, the high-risk early onset component accommodating *VHL* pathogenic variants, and broad higher age component the other known genes (Fig. 2).

## Breast and lung cancer

**Fig. 3:**
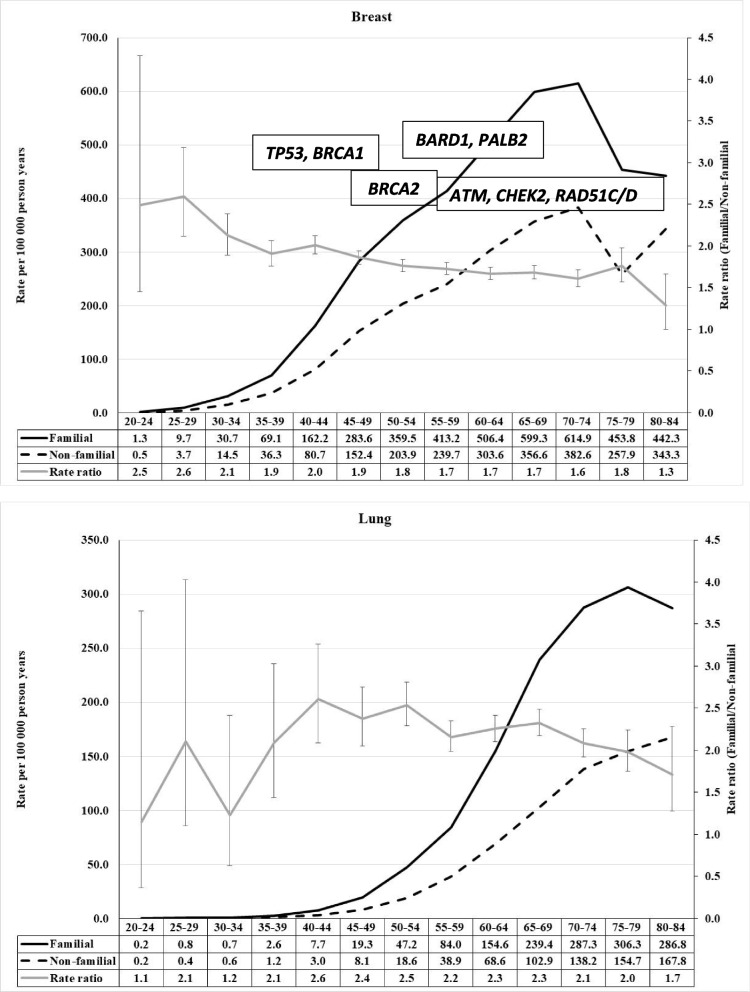
Age-specific incidence rate in population with and without family history of concordant cancer and the rate ratio between the two in breast (upper) and lung (lower) cancers. For breast cancer, most common cancer predisposition genes are also shown with their approximate diagnostic age peak

To keep the figure format, we finally discuss the unrelated cancers of the breast and lung. The proportion of familial cases in breast cancer was 17.5% in FCD [4]. In Fig. 3 we show the age-specific distribution of familial breast cancer. It is characterized by a maximum RR of 2.6 at age 25–29 years and a decrease to 2.0 by age 40–45 years and a plateau at 1.7 towards high ages. The germline genetics of breast cancer was recently described in a comprehensive study on 60,000 cases and 53,000 controls tested by panel sequencing comprising 34 genes [21]. Truncating variants of 10 genes showed a significant (*p* < 0.05) risk in population-based studies, highest for *BRCA1* (10.57), *BRCA2* (5.85) and *PALB2* (5.02). In the same study, in family-based studies, a modified set of truncating variants involving 11 genes was significant, including *PTEN* (OR 11.98) and *CDH1* (6.99), and a lower risk for *BRCA1* (2.77) and *BRCA2* (2.75). Age distribution of mutation carriers was reported in another study for 9 genes [22]. Most cases of *TP53* and *BRCA1* were diagnosed before age 50 years, *BRCA2* cases were diagnosed at age 50 years, most *BARD1* and *PALB2* patients were diagnosed over 50 years of age and *ATM, CHEK2, RAD51C/D* cases were diagnosed well past 50 years of age [22]. In Sweden a large study on women with germline predispositions to breast cancer has been conducted [23]. The most common predisposing genes were *CHEK2* with 160, *BRCA2* with 72, *BRCA1* with 56 and *ATM* with 51 cases but no detailed data were available on diagnostic ages. The mean age at diagnosis of breast cancer in Polish carriers of *CHEK2* mutations was 56 years [24]. Diverse earlier studies on *ATM* have not shown evidence of association with a specific age in breast cancer [25].

The familial proportion of lung cancer was 13.0% [4]. Clustering of age-specific familial risk in lung cancer differs from the previous cancers in showing an early peak at age 25–29 years, and a broad peak at 40–44 years with a highest RR of 2.6 which slowly declined to 1.7 in Fig. 3. Discussing lung cancer without tobacco smoking would be an oversight. RRs for tobacco-related lung cancer are of the order of 10 to 20 in active smokers compared to non-smokers, depending on pack-years smoked, and they remain at levels of 3–5 after 20 years after quitting [26, 27]. Among lung cancer patients diagnosed in Sweden in 2021 only 13% were never-smokers [28]. Nevertheless, Swedish men have been the non-smoking champions in Europe and in the early 2000s their smoking prevalence decreased below the female prevalence [29, 30].

The currently documented hereditary component in lung cancer is not large; germline mutations in *TP53* in the rare Li-Fraumeni syndrome may predispose to lung cancer [31]. In lung adenocarcinoma 27% of the subjects were reported to carry pathogenic germline variants, including *TP53, BRCA2* and other Fanconi anemia genes or other DNA repair genes [32]. Several low-risk genes predispose to lung cancer, most notably the nicotinic acetylcholine receptor (*CHRNA3*) gene variant that modifies smoking dependence and levels [33]. Also variants in metabolic and other low-risk variants may impact lung cancer risk [31]. We have tried to estimate the causes of familial lung cancer based on shared smoking habits and heritability of smoking, and concluded that the risk is a summation of shared smoking habits, environmental sharing and genetic effects [34]. One can assume that shared smoking habits and environmental sharing of smoke intensify familial risk that may explain the peaking of familial risks in middle age. The environmental aspects and preferential selection of smoking spouses are a likely explanation for the spouse correlation in lung cancer [35]. As the causes for familial risk of lung cancer are complex and population-dependent we do not mark any predisposing genes in Fig. 3.

## Conclusions

The first germline susceptibility genes in cancer were found in families that shared certain types of cancers in several generations as a result of Mendelian inheritance. However such families were rare and more recently familial clustering has been ascertained through population databases [4] or systematic diagnostics, such as medical surveillance of familial cancer carried out in the Pomeranian region in Poland [36]. The latter approach has delivered important medical benefits to the affected population globally and secured samples for genetic studies [37]. Here we used the large nation-wide Swedish family set in assessing what age-specific familial risk may tell about the causes of familial cancer. The lung cancer age-specific familial risk profile was distinct from the other cancer profiles in showing the largest familial risk was from middle age to high age as a broad peak which lead us to suggest the contribution by smoking related environmental and inherited causes, as suggested by lacking correlation of cancer between spouses with long cohabitation as discussed earlier [1]. The apparently low contribution of environmental factors to most familial cancers is in no contradiction to the generally recognized main environmental contribution to cancer in general [2, 3]; familial cancer is rare among most cancers [4].

Although we are lacking pathogenic variant confirmation in our assessment, age-related familial risks for CRC, prostate, kidney and breast cancers were consistent with the known germline landscape of these cancers. However, this applied mainly to relatively early onset discrete subsets but not for the older age familial tail which was observed for each cancer and is known from a previous family study considering age of onset [38]. This tail probably accommodates cases that extended sequencing of the older patients has started to detect and may include polygenic inheritance [39]. Such data may question the old wisdom e.g. in Lynch syndrome: “…the Lynch syndrome, is the most common form of hereditary colorectal cancer. Multiple generations are affected with colorectal cancer at an early age (mean, approximately 45 years)…” [40]. With extended follow-up of CRC patients (but only to age 75 years) the median diagnostic age for *MLH1* is about 50 years, *MSH2* over 55 years, *MSH6* over 60 years and *PMS2* over 70 years [7]. However the diagnostics of *PMS2* is complicated because of gene conversion with *PMS2CL* [41].

Going back to data from FCD, we need to remember that even though familial risks are high in early onset cancers, for most cancers the largest proportion of familial cases have been diagnosed at over 70 years of age, with notable exceptions being breast cancer and melanoma [38, 42]. Screening practices may change these proportions over time. Family history was first incorporated in population screening recommendations of breast cancer and CRC prompting an earlier start for screening [43]. Such recommendations have later been included in some other cancers but none of these consider extension of screening to a higher age, based on family history [43]. The present results show that familial risks decline towards high age; however case numbers reached a maximum at around 70 years in breast and prostate cancers and at a higher age for the other cancers. Thus if screening would be considered worthwhile in familial cancers towards higher age there might be a different age optimum depending on the cancer. Among early onset cancers the preset review pointed out an unexplained early onset component for endometrial cancer which probably offers an attractive target for future gene finding efforts.

## Data Availability

No datasets were generated or analysed during the current study.
